# The Fakir Child: Clinical Observation or Invasive Treatment?

**DOI:** 10.3390/pediatric12030023

**Published:** 2020-10-29

**Authors:** Antonio Gatto, Serenella Angelici, Claudia Di Pangrazio, Lorenzo Nanni, Danilo Buonsenso, Filomena Valentina Paradiso, Antonio Chiaretti

**Affiliations:** 1 Institute of Pediatrics, Fondazione Policlinico Universitario A. Gemelli IRCCS, 00168 Rome, Italy; danilo.buonsenso@policlinicogemelli.it; 2 Institute of Pediatrics, Fondazione Policlinico A. Gemelli IRCCS—Università Cattolica Sacro Cuore, 00168 Rome, Italy; sesy93ssj@gmail.com (S.A.); claudiadipangrazio93@gmail.com (C.D.P.); antonio.chiaretti@unicatt.it (A.C.); 3 Division of Pediatric Surgery, Fondazione Policlinico Universitario A. Gemelli IRCCS—Università Cattolica Sacro Cuore, 00168 Rome, Italy; lorenzo.nanni@policlinicogemelli.it (L.N.); filomenavalentina.paradiso@policlinicogemelli.it (F.V.P.)

**Keywords:** foreign body, nail, children

## Abstract

Accidental swallowing of foreign bodies is a common problem among the pediatric population (6 months to 3 years), especially if the foreign body (FB) presents a sharp end that could potentially lead to perforation of the gastrointestinal (GI) tract, resulting in infection and complications. We report the case of a 2-year-old, admitted to the Emergency Department of our hospital after ingesting two FBs classifiable as sharp objects, specifically two metal nails, both approximately 4-cm long, which had been swallowed in one go, as reported by the parents. The patient had been previously admitted to another hospital in the same region, where the Emergency Department (ED) doctors took an X-ray to confirm the ingestion. The foreign bodies ingestion was thus confirmed, and they were, according to their report, located in the GI tract over the stomach. The patient has been monitored through all of his stay in the hospital and the progression of the foreign bodies has been documented with serial X-rays. Since neither clinical nor radiological signs of perforation were present, putting the FBs in the small bowel, a non-operative expectant management was followed. After 4 days of admission, the patient had passed one of the two FBS and later on the second one, without any complication. Thereafter the patient was discharged. The management of sharp gastrointestinal foreign objects ingestion is still debated, and the data of the current literature are poor. A number of case reports and small case series describe successful conservative management for the majority of ingested sharp objects. According to the literature data, our report confirms that the ingestion of sharp objects and relatively big objects in a baby can be successfully non-operatively managed, even despite the age of the patient and though the FBs are multiple.

## 1. Introduction

Foreign body ingestion is a common occurrence among the pediatric population, especially those between 6 months and 3 years of age. As reported by the American Association of Poison Control Centers in 2000, 75% of the >116,000 FB ingestions reported occurred in children aged ≤5 years [1]. A large majority of the foreign bodies in the gastrointestinal (GI) tract are passed spontaneously without complications, although 10–20% of cases require endoscopic intervention, and 1% require open surgery secondary to complications [2]. The nature of the foreign body, its shape, its materials, its dimension and if it is a single object or has been swallowed with other foreign bodies may determine the ability of the foreign body itself to pass spontaneously through the GI tract. The most common foreign body swallowed by children is coins, albeit button batteries, pins, magnets and small parts of toys are also a common occurrence among swallowed foreign bodies [3,4]. The management of sharp gastrointestinal foreign object ingestion is still debated, and the data of the current literature are poor.

We report the case of a 2-year-old, admitted to the Emergency Department of our hospital after ingesting two metal nails, both approximately 4-cm long.

## 2. Case Report

A 2-year-old, Middle Eastern boy was admitted to the Emergency Department (ED) of our hospital after ingesting two foreign bodies (FBs) classifiable as sharp objects, specifically two metal nails, both approximately 4-cm long (approximately 1.57 inches), which had been swallowed in one go, as reported by the parents.

The child had been initially evaluated to another hospital where the X-ray confirmed the ingestion of FBs and location in the GI tract over the stomach. He was then referred to our hospital, where an adequate treatment, consisting of endoscopic exploration and removal of the foreign bodies could eventually be performed. 

**Figure 1:**
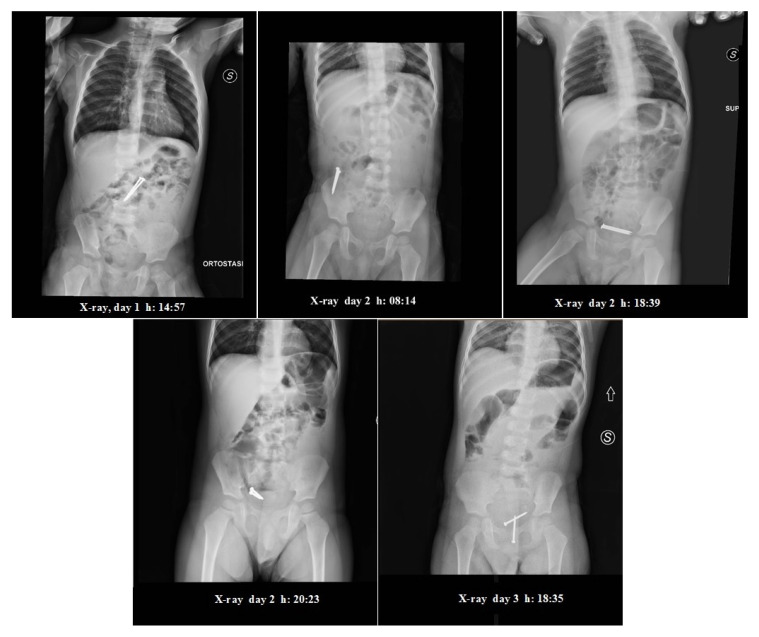
Serial abdominal X-rays.

The first examination assessed that the child was awake, aware and in a stable condition, with no dysphagia or abdominal pain. His abdomen showed no signs of peritonitis. A new X-ray (Figure 1) of the abdomen confirmed the two “nails”—like radiopaque shadows over the ligament of Treitz, with no evidence of free gas under the domes of the diaphragm. Since neither clinical nor radiological signs of perforation were present, putting the FBs in the small bowel, a non-operative expectant management was followed.

Thus, a program of hospital monitoring with a daily abdominal X-ray was assessed and a detailed informed consent of the family was obtained. The patient was encouraged to take Macrogol twice a day, with water or tea.

During the hospitalization, the patient showed no signs of abdominal pain, discomfort or clinical relevance. A new X-ray in supine position (Figure 1) was taken the morning after (2nd day), around 8 am which showed progression of the FBs up to the right iliac region. The FBs kept close proximity to one another. The medical evaluation showed nothing out of ordinary. The patient had not evacuated. Upon consulting with the pediatric surgeon, an enema administration (20/Kg) was performed, after which the patient was allowed to consume liquids (water, tea) and rusks. The patient then evacuated twice, producing normal stools without the FBs in them.

During the late afternoon (2nd day hospitalization), the patient showed sudden signs of distress and discomfort, albeit without vomiting, upon which an X-ray in supine position (Figure 1) was promptly taken, showing the FBs in the pelvic cavity, with a central positioning and one near to another, suggesting their presence in the rectal ampulla. There were no visible signs of free air in the abdomen, and some signs of air fluid levels and dilated loops of bowel.

A rectal exploration was performed with enema administration. In the meantime, the patient had calmed down and showed no further signs of pain or discomfort. Upon consulting with an endoscopist, it was decided not to proceed with a colonoscopy unless the symptoms presented again.

In the following afternoon (3rd day hospitalization), upon assessing that there were no signs of pain or complications, upon presenting a single episode of vomiting in the late afternoon, an X-ray was taken (Figure 1), showing the presence of the two FBs in the rectum, with no signs of air fluid levels nor free air in the abdomen. 

The patient went through the night without showing any sign of complications and, around 1 p.m. on the 4th day, evacuated one of the two FBs. Later on, the patient evacuated the second FB without complications.

The day after (5th day), the patient was clinically evaluated: since he showed no signs of complications or discomfort, the abdomen examination was normal, and the child had resumed feeding. He was deemed suitable for hospital discharge, and was sent home, with the indication of coming back for an outpatient visit in the Pediatric Surgery Ward.

## 3. Discussion

In the Emergency Department, the management of children with a history of ingestion of an FB is relatively common. The diagnosis is often delayed and comes only when symptoms begin to appear. Young children often present only nonspecific symptoms such as refusal to eat, vomiting, wheezing, blood stained saliva or respiratory distress and the diagnosis is very difficult [5,6].

An accurate history is the most important element for a rapid diagnosis and to prevent the complications of FB ingestion. In particular, radiopacity of the objects may prevent the complications reducing the timing of diagnosis.

Most of the time, the foreign body passes spontaneously and without complications, thus requiring no intervention other than monitoring of the patient. However, sharp and pointed objects, such as nails, pins, needles and such, may have a bigger chance to cause serious complications: esophageal ulceration and/or perforation of the GI tract, abscess formation, peritonitis, an aorto-esophageal fistula, and even death [7]. Moreover, sharp objects may get stuck in the GI tract more easily than blunt objects, although this also depends on the size and shape of the object itself.

In clinical practice, management of sharp objects, such as nails in our case, is based on size and location. Therefore, if size is not an issue, these objects do not require endoscopic removal as the trailing sharp ends tend not to perforate [8]. Sites at higher risk of perforation are the duodenal C-loop or ileocecal valve where a long or angulated sharp object may not be able to transit [9].

The majority of studies, on adults and children, have focused on blunt FBs and the management of sharp or pointed FBs is limited to case reports or case series, with rates of surgical intervention from 15% to 35% [10]. Patients requiring surgery had significantly larger objects (6 ± 3 cm) than those who had endoscopy (3 ± 2 cm) or no procedure (2 ± 1 cm) [10].

Gurevich Y et al. in their review on FB ingestion in pediatric patients highlighted the key points for the treatment of ingested sharp object. They recommended obtaining radiographic imaging (which may include CT, MRI, or sonogram if the FB is radiolucent), removing immediately an esophageal sharp object, removing endoscopically gastric sharp objects, and they say that objects wider than 2.5 cm or longer than 6 cm are at higher risk of retention [11].

Cheng W. et al. described 552 children with a proven foreign body with a mean age of 5.2 years. The most common types of foreign bodies, proven radiologically or endoscopically, were coins (49%) and fish bones (29%) [5]. Of 84 children suspected of ingesting metallic objects other than coins, 78 were found by X-ray or endoscopy to still have the foreign bodies in their gastrointestinal tract on admission. Forty-one were classified as sharp objects (pins, needles, screws), 10 removed successfully endoscopically (those lodged at the sites at or proximal to stomach) and 31 mostly lodged in stomach and bowel, at the time of admission, passed out without any complication. The authors demonstrated that radiopacity of the objects may prevent the complications by an early and accurate diagnosis.

Khorana J. et al. in a retrospective study analyzed data on 194 episodes of FB ingestion, with a median age of patients of 43.5 months. The presentation was symptomatic in 55.7% of children and the most common symptom was vomiting (23.2%) followed by dysphagia and abdominal pain [12]. Management included spontaneous passing (60.3%), endoscopy (35.6%), and others (3.1%) [13]. The authors revised also the four most recent guidelines on FB ingestion (NASPGHAN, [14] ESPGHAN, [15] ASGE, [3] Colorado, [16]) and proposed their guidelines. With regard to the esophagus, it was suggested in all cases that esophageal foreign bodies were removed and they recommended the removal of esophageal foreign bodies before 24 h. Regarding FB in the stomach and duodenum, they recommended removing any object longer than 5 cm while the previous guidelines recommended removing 6 cm long objects.

According to current guidelines regarding the ingestion of sharp objects, intervention endoscopy should only be performed if the patient shows any symptoms. If the patient remains asymptomatic, they should be followed clinically with serial X-ray enteroscopy, and surgical removal of the FB should be considered only if they develop symptoms or if the FB has not passed spontaneously after 3 days [9].

## 4. Conclusions

The management of sharp gastrointestinal foreign objects ingestion is still debated, and the data of the current literature are poor. A number of case reports and small case series describe successful conservative management for the majority of ingested sharp objects; however, no known patient or object criteria can be used to predict the outcome. Moreover, even more scarce are data on cases of multiple ingestions of sharp FBs.

According to the literature data, our report confirms that the ingestion of a sharp object in a child can be successfully non-operatively managed despite the small age of the patient and even when the FBs are multiple. Obviously, close clinical monitoring in a hospital setting with daily abdominal X-rays is recommended.

## Abbreviations

GI	gastrointestinal
FB	foreign body
